# Inner Membrane Protein YhcB Interacts with RodZ Involved in Cell Shape Maintenance in *Escherichia coli*

**DOI:** 10.5402/2012/304021

**Published:** 2012-09-03

**Authors:** Gaochi Li, Kentaro Hamamoto, Madoka Kitakawa

**Affiliations:** Department of Biology, Faculty of Science, Kobe University, Rokko, Kobe, Hyogo 657-8501, Japan

## Abstract

Depletion of YhcB, an inner membrane protein of *Escherichia coli*, inhibited the growth of *rodZ* deletion mutant showing that the loss of both YhcB and RodZ is synthetically lethal. Furthermore, YhcB was demonstrated to interact with RodZ as well as several other proteins involved in cell shape maintenance and an inner membrane protein YciS of unknown function, using bacterial two-hybrid system. These observations seem to indicate that YhcB is involved in the biogenesis of cell envelope and the maintenance of cell shape together with RodZ.

## 1. Introduction

Genome analyses predicted that a large number of genes encode cell membrane proteins. However, the majority of them have not well been investigated and remain as function unknown even in *E. coli* [1]. Various genome-wide approaches have recently been taken to examine and obtain clues for defining their function [2–7].

Protein YhcB has been localized to the inner membrane of *E. coli* and is suggested to be a subunit of cytochrome *bd*-type ubiquinol oxidase from proteome studies [8]. However, an investigation by Mogi et al. [9] could not confirm the association of YhcB with this oxidase and its role on assembly and function of the complex is elusive. It was reported that a deletion mutant of *yhcB* could not grow at a critically high temperature [10], but no other phenotype of *yhcB* deletion mutant was found and its cellular function has remained totally unknown. Previously we reported *yhcB* as one of the function-unknown genes that reduce the biofilm formation upon its deletion [11]. In addition, the mutation appeared to cause a synthetically lethal phenotype with the deletion mutation of *rodZ*, indicating a functional relationship between *yhcB* and *rodZ*. A deletion mutant of *rodZ* also exhibited reduced biofilm formation. RodZ is an inner membrane protein and recently it was found to be required for rod-type cell determination [12–14]. Mutants of *rodZ* are nonmotile [11] and show a spherical phenotype similar to mutants of cell shape genes, *mrdAB *and* mreBCD* [15, 16]. However, *rodZ* is not an essential gene and after deletion a cell can divide and continue to grow unlike those of the latter genes. MrdA (PBP2) is an enzyme, elongation-specific transpeptidase, probably working in cooperation with PBP1A [17], while MreB is a bacterial actin homolog that forms helical structures underneath the cytoplasmic membrane. Others are inner membrane proteins and might form the elongase complex, a peptidoglycan (PG) synthesizing machine that elongates the PG side wall [18]. However the mechanism by which they participate in the elongation of PG and whether any other protein is required for the function of this complex is not known. In this study, we further investigated the interaction between *yhcB* and *rodZ* by analyzing the synthetic phenotype and using a bacterial two-hybrid system (BACTH) [19].

## 2. Materials and Methods

### 2.1. General Methods

Bacteria were routinely grown in LB media (0.5% NaCl) at 37°C. Antibiotics ampicillin (100 *μ*g/mL) and kanamycin (40 *μ*g/mL) were added when required. Standard protocols for molecular cloning, transformation, and DNA/protein analyses were used [20]. Plasmids were constructed and purified using strain XL-1blue or DH5*α*. PCR was performed using PrimeStar (Takara Bio Inc. Japan) and genomic DNA of strain KR0401 or relevant plasmid DNA as template following the procedure recommended by enzyme supplier.

### 2.2. *E. coli* Strains and Plasmids

*E. coli* K12 strains and plasmids used in this work are listed in Table 1 together with primers used in PCR amplification. The integration of the inducible *yhcB* gene into chromosome near *attλ*, *λ* (*araC*, *P*_*araBAD*_ *-yhcB, bla*), was performed using *λ*InCh as previously described [14, 21].

In order to analyze the interaction of fusion proteins in the absence of RodZ or YhcB, *ΔrodZ*::*kan* or *ΔyhcB*::*kan* mutations were introduced into JE8471 from corresponding Keio collection mutants [23] by P1 mediated transduction, then kanamycin resistance gene was eliminated through FLP recombination mediated by pCP20 [24]. Plasmid pKnT25 was constructed from pKTop [22] by replacing the region containing *phoA* and *lacZ* fragment of the vector with the fragment coding for T25 using In-Fusion system (ClonTech/Takara Bio). The T25 fragment was amplified by PCR using pKT25 as template and primers, pKTop-T25-5 (GATCCCCGGGTACCGAGCTCGAATTCAATGACCATGCAGCAATCGC) and pKTop-T25-3-rv (AGATTGTACTGAGAGTGCACTTATATCGATGGTGCAGCCCGCCGCGTGCG).

### 2.3. BACTH Analysis

Indicator strain JE8471 harboring indicated plasmids were grown in LB medium containing antibiotics and IPTG (0.5 mM) at 30°C. Cells were collected at a density of about 0.5 OD_600_ and kept frozen at −20°C. Activity of *β*-galactosidase was measured according to Miller [25] except that cells were lysed by adding 0.2% N-lauroylsarcosine (sodium salt) into Z-buffer [26]. Average and standard deviation were calculated from three independent measurements.

### 2.4. Electron Microscopy

Cells were stained by 1% uranyl acetate and observed by TEM as described previously [14].

## 3. Results and Discussion

### 3.1. YhcB Is Required for the Growth of *ΔrodZ* Mutants

**Figure 1:**
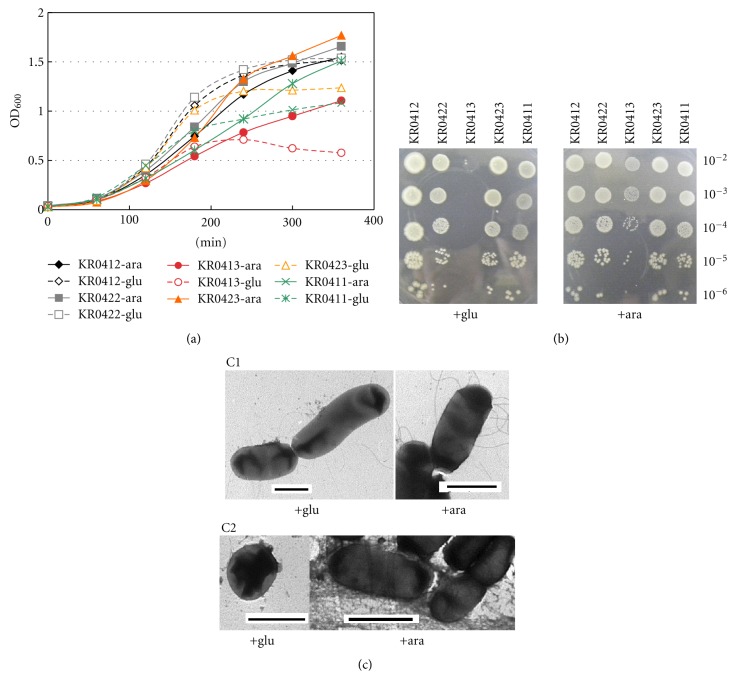
Growth of *ΔyhcB ΔrodZ* mutants carrying an inducible *yhcB* gene. (a) Cells of indicated strains grown overnight in LB medium containing 0.1% arabinose were inoculated to give an OD_600_ of 0.025 into LB medium containing either 0.1% arabinose or 0.2% glucose. Ampicillin was also added at 100 *μ*g/mL to the culture of strain KR0411. Aliquots of culture were taken and OD_600_ were measured at indicated time intervals. KR0412 (*ΔyhcB λ*(*araC*, *p*_*araBAD*_ *-yhcB, bla)*:diamond (black); KR0422 (*ΔrodZ λ*(*araC*, *p*_*araBAD*_ *-yhcB, bla)*:square (gray); KR0413 (*ΔyhcB ΔrodZ:*:*kanλ*(*araC*, *p*_*araBAD*_ *-yhcB, bla)*:circle (red); KR0423 (*ΔrodZ ΔyhcB::kan λ*(*araC*, *p*_*araBAD*_ *-yhcB, bla)*:triangle (orange); KR0411 (*ΔyhcB ΔrodZ::kan*/pBADs-*yhcB*):cross (green). Solid (-) and dotted (⋯) lines indicate the culture with arabinose and glucose, respectively. (b) Strains KR0412 (*ΔyhcB λ*(*araC*, *p*_*araBAD*_ *-yhcB, bla*)), KR0422 (*ΔrodZ λ*(*araC*, *p*_*araBAD*_ *-yhcB, bla*)), KR0413 (*ΔyhcB ΔrodZ::kanλ*(*araC*, *p*_*araBAD*_ *-yhcB, bla)*), KR0423 (*ΔrodZ ΔyhcB::kan λ*(*araC*, *p*_*araBAD*_ *-yhcB, bla)*), and KR0411 (*ΔyhcB ΔrodZ::kan*/pBADs-*yhcB*) were cultured in LB medium containing 0.1% arabinose. Tenfold serial dilutions were made and 5 *μ*L of each were spotted on LB agar plate containing either 0.1% arabinose or 0.2% glucose as indicated, and plates were incubated overnight at 37°C. (c) Cell shape of KR0423 was observed by electron microscopy before (C1) and after (C2) the growth was retarded by YhcB depletion. Horizontal bar is 2 *μ*m.

In our previous investigation we found that the deletion of both *yhcB* and *rodZ* genes caused synthetic lethality because we were unable to introduce the *yhcB::kan* mutation into a *ΔrodZ* strain by P1 transduction [11]. We further examined this phenotype by constructing strains which harbor the *ΔrodZΔyhcB* double deletion mutation and an inducible *yhcB* gene either on a plasmid or integrated in the chromosome, and monitoring the cell growth under *yhcB*-induced or repressed conditions. As shown in Figure 1(a), the growth of strain KR0413 (*ΔyhcB ΔrodZ::kan P*_*araBAD*_-*yhcB*) was retarded when the cell density was about OD_600_ of 0.7 after 4.2 generations in a medium containing 0.2% glucose, whereas it continued beyond OD_600_ of 1.1 in the presence of 0.1% L-arabinose. The growth inhibition in the presence of glucose was also observed on agar plates (Figure 1(b)). In the case of strain KR0411 that carried *P*_*araBAD*_-*yhcB* gene on a multicopy plasmid, cells continued to grow more than 5 generations under the repressed condition and the cell density reached over 1.1 OD_600_. This might indicate that KR0411 probably contained more YhcB molecules than KR0413 and it took more generations under the repressing condition until the amount of YhcB in cell dropped to a critical level for the cell growth. On agar plates, this strain as well as KR0423 (*ΔrodZ ΔyhcB::kan P*_*araBAD*_-*yhcB*) showed similar growth on L-arabinose and glucose medium (Figure 1(b), lane 4 and 5). The reason for this discrepancy is not clear but we assume that the effect of *ΔrodZ* mutation on the cell is larger in liquid culture than on solid agar plates, probably because *rodZ* is important for PG synthesis [14] and cells with a defect in PG might not well resist the turgor pressure in a liquid environment. We often observed *ΔrodZ *mutants lyse when cultured in liquid medium for a long period. KR0412 (Δ*yhcB P*_*araBAD*_-*yhcB*) and KR0422 (Δ*rodZ P*_*araBAD*_-*yhcB*) as well as wild type cells (data not shown) grew similarly in the medium containing L-arabinose and glucose, which clearly showed that single mutation, either *ΔyhcB* or *ΔrodZ*, does not affect the growth in these conditions.

We previously reported [14] that *ΔrodZ* mutants which are nonmotile often changed phenotype probably by acquiring a mutation(s) spontaneously. During the construction of double deletion strain starting from a *ΔrodZ* mutant a similar process seemed to have happened. The resultant strain KR0423 (*ΔrodZ ΔyhcB::kan P*_*araBAD*_-*yhcB*) was found to be motile and grow much faster than KR0413 (Figure 1(a)). Interestingly, the growth of this strain seemed to be still dependent on the expression of the *yhcB* gene, though the growth continued more than 5 generations and the cell density reached to 1.2 OD_600_, about two fold of KR0413 in the repressing condition. Furthermore, the cell shape of KR0423 was found to be rod-type rather than spherical as KR0401*ΔrodZ*-mot^+^, a previously reported motile derivative of KR0401*ΔrodZ* mutant, and however KR0423 showed again a spherical phenotype when it was grown in the medium containing glucose and YhcB depleted (Figure 1(c)). This seems to further indicate that YhcB also participates in lateral cell growth by interacting with RodZ and probably with other components involved in cell morphogenesis.

### 3.2. YhcB Interacts with RodZ

**Figure 2:**
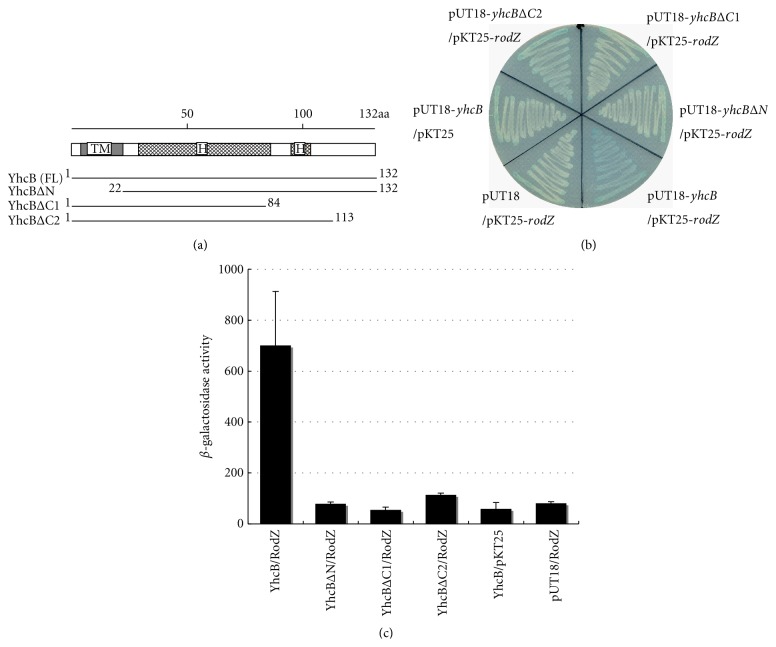
Interaction of YhcB and RodZ detected by BACTH system. (a) Schematic representation of YhcB and its truncated derivatives. Predicted transmembrane domain (TM) and cytoplasmic helical structures (H) are indicated. Thick lines with numbers of amino acid residues show regions cloned into indicated plasmids. (b) Indicated plasmids were cotransformed into JE8471 and resultant transformants were streaked on a LB indicator plate containing X-gal (40 *μ*g/mL) and IPTG (0.5 mM), and incubated at 30°C for one day. Blue colour indicates a positive interaction between each fusion protein. (c) Interaction in each cotransformants was also examined quantitatively by *β*-gal assay as described in Section 2.

Because the synthetic phenotype described above indicated a functional interaction between YhcB and RodZ, we investigated whether they associated *in vivo* using a BACTH system [19]. In this system, two complementary fragments T18 and T25 derived from the adenylate cyclase of *Bordetella pertussis* are fused with proteins of interest, expressed in a *cyaA* (adenylate cyclase) mutant strain and the activity of *lacZ* is examined. In *E. coli*, cyclic AMP bound to the transcriptional activator, CAP, triggers transcriptional activation of catabolic operons, such as lactose. Therefore, in *ΔcyaA* strain (JE8471) the synthesis of cyclic AMP by the reconstructed adenylate cyclase of *B. pertussis* is required for the expression of *lacZ*. We first constructed YhcB-T18 and T25-RodZ fusions using plasmid pUT18 and pKT25, respectively, because it was predicted that both proteins have a single transmembrane domain and C-terminus of YhcB and N-terminus of RodZ are located in the cytoplasm [1]. The result on the indicator plate showed that indeed YhcB physically interacted with RodZ. Next, we investigated which part of YhcB is required for the interaction using deletion mutants of *yhcB*. As shown in Figure 2, YhcBΔN (amino acid residues 22–132) and YhcBΔC1 (1–84) did not show a detectable interaction but YhcBΔC2 (1–113) weekly interacted, indicating that the transmembrane domain (TM) and the cytoplasmic helical structures are required and the extreme C-terminal region predicted to have no secondary structure [27] is also important for interaction with RodZ. However, Western blot analysis of fusion proteins in cells harboring plasmids exhibited that protein amounts of deletion mutants are significantly reduced (data not shown), indicating that these truncated YhcB fusions were unstable and this could be the reason of observed low *β*-galactosidase activity. Alternatively, the absence of interaction might have led to the degradation of unincorporated proteins. In any event, the C-terminal region of YhcB seemed to be important for its interaction with RodZ. On the other hand, the helix-turn-helix domain (amino acid residues 30–49) of RodZ required for proper function of RodZ [14] was dispensable for the interaction (data not shown).

### 3.3. YhcB Interacts with Cell Shape Proteins

Because RodZ is important for rod-type cell determination and interacts with proteins involved in cell morphogenesis [13], we further examined whether YhcB also interacts with these proteins and MurG, an enzyme in the pathway of peptidoglycan synthesis. In order to perform BACTH analysis, *mreB*, *mreC*, *mreD*, and *murG* were cloned into pKT25 and *rodA* into pKnT25, respectively, and *β*-galactosidase activities of transformants carrying one of these constructs and pUT18-*yhcB* were measured. The results obtained indicated that YhcB interacts with MreC, MreD, RodA, and MurG, but not with MreB (Table 2).

**Figure 3:**
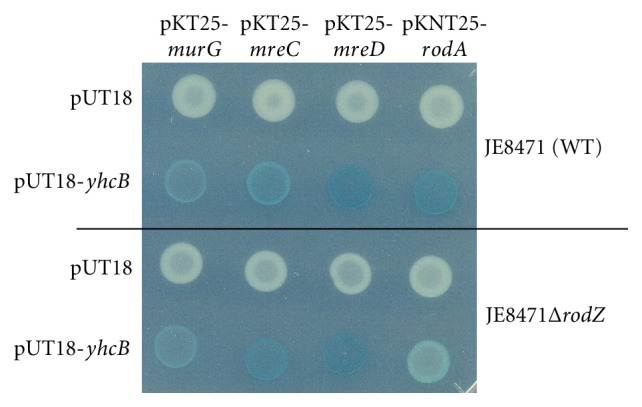
Interactions of YhcB and shape proteins are RodZ independent. Indicated plasmids and pUT18-*yhcB* or pUT18 vector were cotransformed into JE8471 (upper) or JE8471Δ*rodZ *(lower) and interactions were examined as described in Figure 2(b).

Here, it was notable that unlike RodZ that seems not to interact with MurG and other peptidoglycan synthesizing enzymes [13, 28], YhcB showed a significant interaction activity with MurG. On the other hand YhcB seems not to interact with cytoskeleton protein MreB. In addition, the interactions were studied in a Δ*rodZ* derivative of indicator strain JE8471 to investigate whether the association of YhcB with these cell shape proteins is independent on the presence of RodZ. As shown in Figure 3, interactions with these cell shape proteins were also detected in the absence of RodZ (JE8471Δ*rodZ*), though the interaction with MurG seemed to be weaker compared to the wild type strain. This might indicate that RodZ somehow participates in the interaction between YhcB and MurG, for example, by stabilizing their association. However, the overproduction of RodA-T25 and T25-MurG fusion proteins in *ΔrodZ* mutant could be deleterious to the cell, because about 60% of transformants were blue but the rests were nearly white, and a significant number of these could not grow upon single-colony purification. This could be the reason why the color of pKT25-*murG* transformants of JE8471*ΔrodZ* was lighter than that of JE8471. In the white cells, T25-MurG fusion protein was probably not produced.

We also investigated whether YhcB interacts with CydB, a component of cytochrome *bd*-I oxidase, and a cell division protein FtsB. No apparent interaction was observed between YhcB and CydB, which further supported the conclusion by Mogi et al. [9] that YhcB is dispensable for the assembly and function of cytochrome *bd*-1 oxidase. FtsB, a divisome assembly protein [29], likewise showed no detectable activity with YhcB, indicating that YhcB does not associate with divisome nor merely interact with proteins anchored in IM (inner membrane).

### 3.4. Possible Function of YhcB

Recent global analysis of interacting proteins [7] indicated that YhcB associated with YciS, an IM protein of unknown function. Therefore, we next examined this using BACTH system. YciS has two TM domains and both N- and C-termini of the protein were predicted to be cytoplasmic [30]. Therefore, YciS was cloned into pKT25 and interaction with YhcB-T18 was examined. The result showed that they indeed interacted (Table 2). In addition, YciS was found to show a significant interaction with RodZ and this interaction was independent of YhcB because similar *β*-galactosidase activity was shown in *ΔyhcB* indicator strain (data not shown). Furthermore, YciS showed a significant interaction with FtsB (data not shown). Though further examination is necessary, intriguingly this might indicate that YciS participates both in lateral elongation of peptidoglycan and septum formation. Finally, it was predicted that YhcB forms a homooligomeric complex [31]. Our BACTH analysis supported this by detecting a strong self-interaction (Table 2).

Taking together all interactions detected by BACTH, we speculate that the complex integrated in IM consisting of YhcB, RodZ, and YciS together with cell shape proteins MreCD and RodA assists PG synthesis by directing enzymes and other molecules required at the site of PG synthesis. Probably they primarily function in the cell elongation but might also participate in septum formation and therefore the more severe defect caused by the simultaneous loss of RodZ and YhcB inhibited the cell growth.

## 4. Conclusion

We showed the synthetic lethality of *yhcB* and *rodZ* deletion mutations by constructing a strain that harbors double deletion mutations and carries an inducible *yhcB* gene on the chromosome. Furthermore, using BACTH system, the YhcB protein was shown to interact with cell shape proteins RodZ, MreCD, and RodA, an enzyme required for PG synthesis, MurG, and a function-unknown YciS that also interacted with RodZ.

Because the loss of YhcB showed neither a growth defect nor apparent phenotype in general culture conditions, the function of YhcB has remained obscure. However, our investigations seemed to indicate the involvement of this protein in cell shape determination and/or the biosynthesis of peptidoglycan cooperating with RodZ. Recent extensive investigations identified a large number of proteins involved in the murein synthesis and cell morphogenesis [18, 32]. However, it is still not clear how these proteins are organized and function in IM, whether they participate also in the septum formation and what is the mechanism to regulate the cell elongation and cell division. Further investigation of YhcB and RodZ as well as of YciS would give more insights into the elaborated mechanisms of cell envelope biosynthesis and its regulation. These proteins could also be the targets of new antimicrobial drugs, because the biofilm formation is a critical factor in pathogenicity [33, 34] and the loss of these proteins caused reduced biofilm formations.

## Figures and Tables

**Table 1:** *E. coli* strains and plasmids used in this study.

Strain	Genotype^a^	Source or reference
XL-1Blue	F′ [*proAB^+^, lacIq, lacZDM15::Tn10 (tetr)*]*, hsdR17, *	Laboratory stock
	*supE44, recA1, endA1, gyrA46, thi, relA1, lac*	
DH5*α*	F^−^, *deoR, endA1, gyrA96, hsdR17(rk^−^mk^+^), recA1, *	Laboratory stock
	*relA1*, *supE44, thi-1, D (lacZYA-argF)U169, *(*f 80lacZ D M15*)	
JE8471	*DE(cya)854, trp, his, ilv *	National BioResource Project
BW25113	F^−^,* rph-1, DE(rhaBAD)568, DE(araBAD)567, *	CGSC
	*DElacZ4787, hsdR514, rrnB*	
KR0401	a derivaative of BW25113	[11]
KR0411	KR0401Δ*yhcB/*pBADs-*yhcB *	This study
KR0412	KR0401 Δ*yhcB*, *λ*(*araC, p*_*araBAD*_ *-yhcB, bla) *	This study
KR0413	KR0412 Δ*rodZ::kan *	This study
KR0422	KR0401 Δ*rodZ*, *λ*(*araC, p*_*araBAD*_ *-yhcB, bla) *	This study
KR0423	KR0422Δ*yhcB::kan *	This study

plasmid	Relevant features or primers used to amplify the indicated gene	

pKT25	BACTH vector, Km^R^	[22]
pKnT25	constructed from pKTop, Km^R^	This study, [22]
pUT18	BACTH vector, Ap^R^	[22]
pUT18c	BACTH vector, Ap^R^	[22]
pKnT25-*rodA *	ctctagaGACGGATAATCCGAATAAAAAAA/	This study
	gcggtaccACGCTTTTCGACAACATTTTCC	
pKnT25-*yhcB *	*yhcB* derived from pUT18-*yhcB *	This study
pKT25-*cybD *	gctctagaGATCGATTATGAAGTATTGCGT/	This study
	gcggatccTACAGAGAGTGGGTGTTACGTT	
pKT25-*mreB *	gctctagaGTTGAAAAAATTTCGTGGCATG/	This study
	cgggatccTCTTCGCTGAACAGGTCGCCG	
pKT25-*mreC *	gctctagaGAAGCCAATTTTTAGCCGTGG/	This study
	gcggtaccTGCCCTCCCGGCGCACGC	
pKT25-*mreD *	gctctagaGGCGAGCTATCGTAGCCAGGGA/	This study
	gcggtaccTGCACTGCAAACTGCTGACGGA	
pKT25-*murG *	gctctagaGAGTGGTCAAGGAAAGCGATTA/	This study
	gcggtaccGCCCGGGCAACCCGGCTCACTT	
pKT25-*rodZ *	*rodZ* derived from pUT18c-*rodZ *	This study
pKT25-*yciS *	gctctagaGAAATATTTACTCATTTTCTTACTGGT/	This study
	gcgaattcTTATTCCTTCGCCGCTGAC	
pUT18c-*rodZ *	gctctagaGAATACTGAAGCCACGCAC/	This study
	ggaattCTGCGCCGGTGATTGTT	
pUT18-*ftsB *	gcctgcag GGGTAAACTAACGCTGCTGTTG/	This study
	cgggatcc CGATTGTTTTGCCCCGCAGA	
pUT18-*yhcB *	gcctgcag GACCTGGGAATATGCGCTAATT/	This study
	cgggatcc TCGCGCTTCGCGCCAGTA	
pUT18-*yhcB*Δ*C1 *	gcctgcagGACCTGGGAATATGCGCTAATT/	This study
	cgggatccAGGCTGCTGGAGCTTTTTGC	
pUT18-*yhcB*Δ*C2 *	gcctgcagGACCTGGGAATATGCGCTAATT/	This study
	cg*ggatcc*ATCTGCACCGGTGCCTGATCGT	
pUT18-*yhcB*Δ*N *	gc*tctaga*GCGTTTTGGTAATCGTAAACTAC/	This study
	cgggatccTCGCGCTTCGCGCCAGTA	

^a^ *λ*(*araC ParaBAD-yhcB*) denotes chromosome integration mediated by *λ*InCh.

**Table 2:** BACTH analysis of interaction between YhcB and inner membrane proteins.

	*β*-gal activity^a^	
T25-plasmid^b^	YhcB-T18	T18
T25-MurG	269.6 ± 60.7	71.4 ± 5.5{}{}{}
T25-MreB	68.0 ± 9.2	73.7 ± 9.2
T25-MreC	476.6 ± 3.0	71.2 ± 6.3
T25-MreD	254.5 ± 52.4	75.6 ± 22.5
RodA-T25	379.7 ± 19.1	82.7 ± 18.6
T25-RodZ	701.1 ± 211.7	80.6 ± 6.1
T25-CydB	87.3 ± 27.8	70.9 ± 10.9
T25-YciS	853.4 ± 685.6	78.2 ± 2.1
T25	59.3 ± 24.4	31.1 ± 21.6

T18-plasmid^c^	YhcB-T25	T25

T18-RodZ	294.5 ± 14.2	78.0 ± 7.9
T18-FtsB	82.7 ± 6.3	64.9 ± 14.1
YhcB-T18	1236.8 ± 398.2	59.3 ± 24.4

Interaction between YhcB and indicated proteins were quantified by measuring *β*-galactosidase activities of transformants harboring the corresponding plasmids.

^a^Numbers indicate averages of *β*-galactosidase activity (Miller's unit) with standard deviations. T18 and T25 show vector only.

^b^Plasmids with T25-CyaA domain appended to N (T25-) or C (-T25) terminus of indicated proteins.

^c^Plasmids with T18 CyaA domain appended to N (T18-) or C (-T18) terminus of indicated proteins.
